# Upper limb performance across temperature extremes: evaluating functional risks in carpal tunnel syndrome and healthy workers

**DOI:** 10.3389/fspor.2025.1619540

**Published:** 2025-12-16

**Authors:** Shahed Obeidat, Mustafa Rawshdeh, Bandar Alzahrani, Moayad Tanash, Abdullah Al-Hjjaji

**Affiliations:** 1 Department of Industrial Engineering, The Jordan University, Amman, Jordan; 2 Department of Industrial Engineering, The Hashemite University, Zarqa, Jordan; 3 Department of Industrial Engineering, College of Engineering in Yanbu, Taibah University, Yanbu Governorate, Saudi Arabia; 4 Department of Industrial Engineering, Umm Al-Qura University, Makkah, Saudi Arabia

**Keywords:** manual dexterity, surface electromyography (SEMG), carpal tunnel syndrome (CTS), ambient temperature, hand grip strength (HGS)

## Abstract

**Objective:**

This study explores the effect of exposing the upper limb to different levels of temperature and compares results between healthy humans and humans with Carpal Tunnel Syndrome (CTS). Additionally, gender disparities are addressed in the study.

**Background:**

Muscle efficiency is affected by ambient conditions. Awareness of such conditions increase work efficiency, avoid excessive muscle strain and prevent performance variability.

**Method:**

A full factorial design was used to recruit 12 participants for the study, six of whom were diagnosed with CTS. The procedure included three stages: an initial 15 min cooling phase in 10 °C water, a 15 min warming in 35 °C water, and a final recovery phase at room temperature (∼25 °C). At each stage, muscle dexterity and strength were assessed using three tests, namely, gross motor skills test, fine motor skills test and hand grip strength (HGS) test.

**Results:**

Changes in ambient temperature were found to have a positive significant effect on human functionality in terms of dexterity and strength. According to the experimental runs, males functionality showed more variation across different temperature levels compared to that of females. Gender appeared as a significant factor in performance of participants at the gross motor skills test but not the fine motor skills test. CTS diagnosed participants exhibited a significant variation in the performance of fine motor skills but not in the gross motor skills when compared to healthy participants.

**Conclusion:**

Keeping the upper limb in warm temperature is critical to maximize performance of workers and prevent discomfort and injuries.

## Introduction

Manual dexterity is a measure of the upper limb performance to precisely perform tasks with arm, hand, and fingers. Dexterity includes fine motor skills and gross motor skills which usually require eye-hand coordination or hand-arm coordination (1). Studies on manual dexterity have shown that the performance of the arms, hands, and fingers can be influenced by factors, such as gender, musculoskeletal disorders, and body temperature. Exposure to extreme temperatures, even for a short time, can directly affect skin temperature, which may eventually reduce the manual dexterity of the upper limb. Change in manual dexterity can be seen in form of the total time required to complete the task, maximum muscle contraction, or time to the onset of fatigue (2–5).

Cold work environments or manual handling of cold objects have been shown to reduce upper limb performance, which can be attributed to the narrowing of blood vessels caused by low temperatures. This forces the heart to work harder to supply blood to the muscles. Reduced blood circulation under cold exposure decreases oxygen delivery and slows nerve conduction, leading to weaker and less efficient muscle contractions. On the contrary, high temperature levels are generally associated with improved worker performance and increased muscle strength. Studies on animals have been conducted to examine the muscle efficiency as a function of heat and work at different temperature levels. The results showed trends in muscle efficiency similar to those observed in human experiments (5–8).

Carpal tunnel syndrome (CTS) is a neurological disorder where the median nerves extended from the forearm to the fingers, can experience severe compression as they passes through the carpal tunnel at the wrist. This condition results in discomfort, pain, and reduced dexterity of the upper limb. Maximum CTS symptoms are seen at the first three fingers (thumb, index, and middle fingers). CTS is diagnosed up to ten times more often in females than in men, and female patients typically report more severe symptoms (9–11). Individuals diagnosed with CTS, who already experience compression of the median nerve at carpal tunnel, are particularly affected by cold weather. Low temperatures cause additional constriction of blood vessels throughout the body, slowing circulation and reducing blood flow to the upper limbs. This can lead to increased sensitivity to pain, a higher risk of injury, and localized inflammation where prolonged or repeated exposure to cold can impair local circulation, leading to ischemia reperfusion stress and mild inflammatory responses in muscular and neural tissues, which may further compromise upper limb performance (12). In general, humans tend to move less in cold weather. Reduced physical activity can further slow blood circulation, worsening the symptoms of CTS. Additionally, synovial fluid in the joints which enables smooth movement can thicken in low temperatures, further limiting wrist mobility in individuals with CTS. Cold weather may also impact muscle tissue by increasing air pressure, causing the tissues to expand and place additional pressure on the median nerve at the wrist (13–17).

However, multiple studies have demonstrated that heat therapy effectively improves CTS symptoms. It has been clinically recognized as a viable alternative to surgical procedures for significantly reducing symptom severity, improve manual dexterity, and alleviating pain in patients with mild to moderate CTS. Additionally, heat therapy helps avoid the side effects associated with invasive medical treatments (18, 19).

### Research gap

Prior studies have examined the effects of temperature, gender, and musculoskeletal conditions independently, limited research has explored their combined impact on upper limb performance. In particular, the interaction of these variables on both fine and gross motor function, as well as muscle activation patterns, remains under-investigated. This study addresses this gap by employing sEMG to explore the effects of ambient temperature, gender, and carpal tunnel syndrome on dexterity and strength.

### Application

The findings offer practical implications for occupational health, particularly in thermally variable work environments. Enhancing thermal conditions, especially warming the upper limbs, may improve manual performance and reduce strain, with notable benefits for individuals with CTS. These insights support the development of ergonomic interventions, such as task-specific temperature controls and preventive strategies tailored to worker health status and gender.

### Method

Fingers and hand dexterity were assessed using two separate tests: fine motor skills test measured with O'Connor Tweezer Dexterity Test (Lafayette Instrument Model 32022) and gross motor skills tests measured with Minnesota dexterity test (Lafayette Instrument Model 32023A). Forearm strength was measured using a dynamometer (Lafayette Hand Dynamometer Model J00105) and surface electromyography (Biometrics ltd). The procedure for measuring dominant-hand grip strength (HSG) followed the protocol established by Shih (2007).

Before the experiment began, participants were introduced to the instruments, the intended outcomes, and the detailed procedure. A consent form outlining the study goals and procedure was distributed and signed by the participants. Data were analyzed using DATAtab 2025 and MATLAB 2019b, and the results were compared between the healthy group and the CTS-diagnosed group.

### Participants

A total of 12 participants were recruited for this study (see the experimental design section for additional details). The participants were aged between 18 and 25 years. Of the total, Six had no history of musculoskeletal disorders, while six had been previously diagnosed with carpal tunnel syndrome (CTS) and were currently experiencing mild to severe symptoms. All participants wore short-sleeved laboratory coats during the session, and demographic data were collected as shown in Table 1.

**Table 1:** Demographic statistics.

Group	Healthy	CTS-diagnosed	Total	Variable	Mean	S. D
Male	3	3	6	Age	24.5	3
Female	3	3	6	Weight	80.3	6.6
Total	6	6	12	Stature	170.3	5.3

### Procedure and data collection

#### Pilot test and pretest

**Figure 1:**
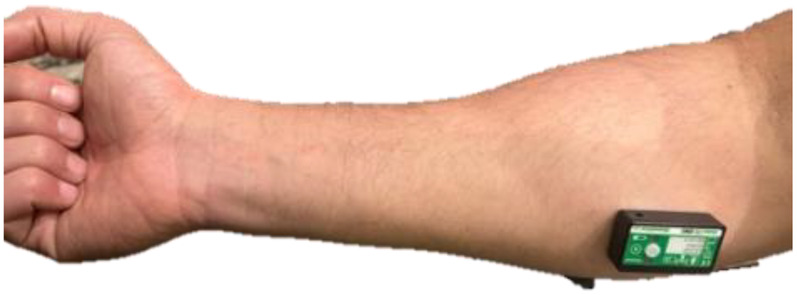
Forearm sEMG sensor (K23866) installed at the belly of the inner forearm muscle while fully contracted.

To minimize potential learning effects on the experimental outcomes, a pilot study was conducted with five individuals who were not part of the main study sample. Each participant in the pilot completed 10 trials of both O'Connor Tweezer Dexterity Test and Minnesota dexterity test. Results were analyzed using one-way analysis of variance (ANOVA), which revealed a significant improvement in performance up to the 5th trial. Beyond the fifth trial, no significant change was observed. Based on these findings, all 12 participants in the main study completed five practice trials on both instruments prior to the formal testing procedures. A forearm sensor, installed according to the manufacturer's instructions (Biometrics Ltd.), (Figure 1) was used to monitor muscle activity (i.e., electrical signals) during the experimental procedures. To insulate the surface Electromyography (sEMG) sensors and slow heat transfer during water immersion, elbow length latex gloves and tape were applied. These were removed prior to each task to eliminate any influence on participant performance.

In the first stage, all measurements were taken at room temperature (∼25 °C). Each participant began with the Minnesota Dexterity Turning test, followed by a 15 min rest period. This was followed by the O'Connor Tweezer Dexterity Test, then a 15 min rest, and finally the HSG test performed in three positions: neutral, pronation, and supination. sEMG sensors were activated at the beginning of the HGS test to record muscle activity throughout.

**Figure 2:**
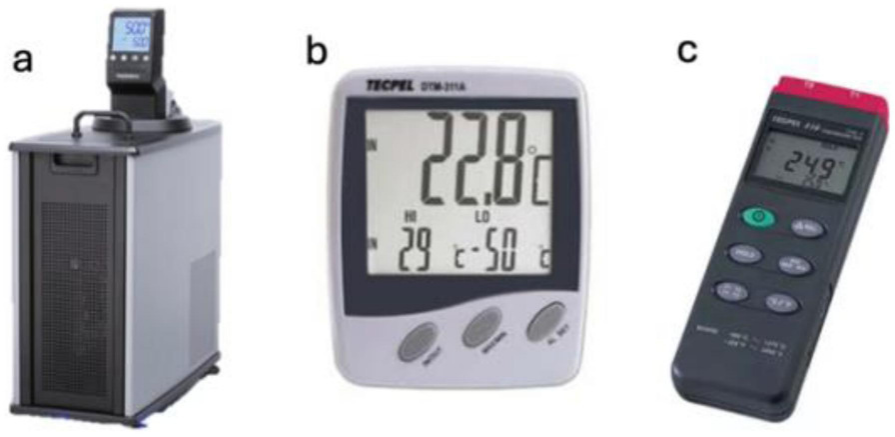
Temperature control apparatus. **(a)** Polyscience® Water Circulators/REFRIGERATED CIRCULATOR 7 LITER, MX, −20° C. Temperature range −20°–135°. Temperature stability ±0. 07°. Timer range 0 to 9,999 min/hr. Overall Dimensions (L × W × D) (cm) 54.1 × 22.1 × 64.5 cm. Working Access (L × W × D) (cm) 25 × 31.6 × 14 cm. **(b)** Digital thermometer and hygrometer made by TECPEL (Model DTM301). Temperature range −10 °C–+50 °C. resolution 0.1 °C. Humidity: 20%–99%, with 1% resolution. **(c)** A digital four-channel thermometer made by TECPEL (Model DTM319). Measuring Range −200–1370 °C (−328–2,498°F). Accuracy: ±(0.3% + 1 °C) at −200 °C–200 °C. Resolution: 0.1 °C. Type of Thermocouple: K-Type. Sampling rate: 3 s per testing circle.

The second stage involved a cooling procedure, during which participants immersed their dominant upper limb (from fingers to elbow) in a 10 °C water bath for 20 min. To control the water temperature, ambient conditions, and skin temperature, the following apparatus were used (see Figure 2).

Immediately after 20 min, each participant began with the Minnesota Dexterity Turning test, and sEMG attached sensors were set to record (forearm muscle activity, wrist range of motion, and task completion time). Next, the participant performed another immersion in a 10 °C water bath for 15 min to return to the required skin temperature (10 °C). Just after the stipulated time, the participant was directed to the working station (an ergonomic adjustable chair and table, preset to suit participant's anthropometrics) to perform HSG using a hand Dynamometer in neutral, pronation, and supination positions. After a final 15 min immersion, the participant began with the O'Connor Tweezer Dexterity Test, and sEMG sensors were again set to record (forearm muscle activity, wrist range of motion, and task completion time).

The third stage was the warming phase, where participants soaked their dominant upper limb (from fingers to elbow) in a 35 °C water bath for 15 min. The same set of temperature control apparatus was used as in the previous stages. The soaking periods differed because it takes the body less time to warm than to cool. The same procedure from stage two was repeated, starting with the Minnesota test, followed by 10 min of immersion, the O'Conner tweezer test, another 10 min of immersion, and concluding with the HGS test in the three different positions.

The three temperature conditions were specifically chosen to represent a spectrum of thermal exposures commonly encountered in occupational settings, particularly those involving manual labor and fine motor tasks.

### Experimental design

A full factorial design was used to assess the relationship between three independent variables: Gender with two levels male and female, temperature with three levels cool, room temperature, and warm, and health with two levels healthy and CTS-diagnosed. Three responses were assessed; fine motor skills measured in min, gross motor skills measured in min, and strength measured in Lbs. Main effect and interaction effects were assessed.

## Results

A separate full factorial design model was created for each of the three responses (fine motor skills, gross motor skills, and strength). The three models were analyzed using DATAtab software to explore main effects and two-way interactions. Table 2 provides the descriptive statistics of DOE responses.

**Table 2:** Descriptive statistics.

Response	Independent variable level	Mean	Std. deviation	Minimum	Maximum
Avg. MVC (LB)	Cool	63.5	23.1733	38	86
	Room temp.	71.75	31.4576	36	102
	Warm	80.75	39.66	36	118
Gross motor skills (min)	Cool	1.6925	0.0727	1.62	1.79
	Room temp.	1.5475	0.1537	1.4	1.69
	Warm	1.39	0.1817	1.2	1.56
Fine motor skills (min)	Cool	2.4075	0.3806	1.92	2.77
	Room temp.	2.2425	0.4186	1.8	2.6
	Warm	2.11	0.4746	1.7	2.56
Avg. MVC (LB)	Healthy	78.3333	27.9762	50	118
	CTS diagnosed	65.6667	33.1642	36	110
Gross motor skills (min)	Healthy	1.5367	0.1684	1.27	1.7
	CTS diagnosed	1.55	0.2129	1.2	1.79
Fine motor skills (min)	Healthy	1.8983	0.226	1.7	2.3
	CTS diagnosed	2.6083	0.096	2.48	2.77
Avg. MVC (LB)	Male	98.3333	14.4453	80	118
	Female	45.6667	10.2892	36	59
Gross motor skills (min)	Male	1.43	0.1835	1.2	1.66
	Female	1.6567	0.0963	1.53	1.79
Fine motor skills (min)	Male	2.3033	0.403	1.7	2.77
	Female	2.2033	0.4408	1.7	2.64

### Fine motor skills

The O'Connor Tweezer Dexterity Test was used to evaluate participants’ fine functional dexterity. Since the number of runs was small, Shapiro–Wilk normality test was conducted at a 95% confidence level to verify the normality assumption. The Shapiro–Wilk test yielded a *p*-value of 0.066, indicating that the model data does not significantly deviate from normality (20).

Analysis of variance (ANOVA) results in Table 3 indicate a high significant effect of the model on the measured response. Both temperature and health have significant influence on fine motor skills with health being a strong predictor (F = 528.44) of total time required to complete the task. However, gender had no significant effect (*p* = 0.12) on fine motor skills which suggests no differences in performance between male and female.

**Table 3:** ANOVA for the fine motor skills model.

Variable	df	Adj SS	Adj MS	F	*p*
Model	9	1.75	0.19	69.27	.014
Temperature	2	0.15	0.07	25.94	.037
Health	1	1.48	1.48	528.44	.002
Gender	1	0.02	0.02	6.84	.12
Temperature*health	2	0.02	0.01	3.78	.209
Temperature*gender	2	0.06	0.03	11.23	.082
Health*gender	1	0.02	0.02	6.28	.129
Error	2	0.01	0		

Fine functional tests employ small hand and finger muscles to work with the help of eye-hand coordination to perform the task in an efficient manner, regardless of the muscle mass and strength. Hence, no clear gender-based variation was observed. On the other contrary, there was a noticeable difference between the performances of Carpal Tunnel Syndrome (CTS)-diagnosed participants and healthy participants, since CTS symptoms include lack of hand and fingers coordination along with weakness in wrist.

Table 4 presents the estimated coefficients, indicating the direction and strength of the relationships between the factors and the response variable. Room temperature decreased the time required to complete the task by 0.16 min, while increasing the temperature to attain a warmer environment further decreased the time by 0.27 min. Health factor was found to have positive estimated coefficient of 0.3, meaning that CTS diagnosed participants are expected to finish the task by an additional 0.3 min compared to that required by healthy participants (assuming same levels of temperature and gender).

**Table 4:** Estimated coefficients for fine motor skills model.

Model	Coefficients	Standard error	t	*p*
Constant	2.41	0.03	90.86	<.001
Temperature_room temp.	−0.16	0.04	−4.4	.048
Temperature_warm	−0.27	0.04	−7.14	.019
Health	0.3	0.03	11.23	.008
Gender	−0.13	0.03	−4.81	.041
Temperature_room temp.*health	0.06	0.04	1.6	.25
Temperature_warm*health	0.1	0.04	2.74	.112
Temperature_room temp.*gender	0.09	0.04	2.27	.151
Temperature_warm*gender	0.18	0.04	4.74	.042
Health*gender	0.04	0.02	2.51	.129

**Figure 3:**
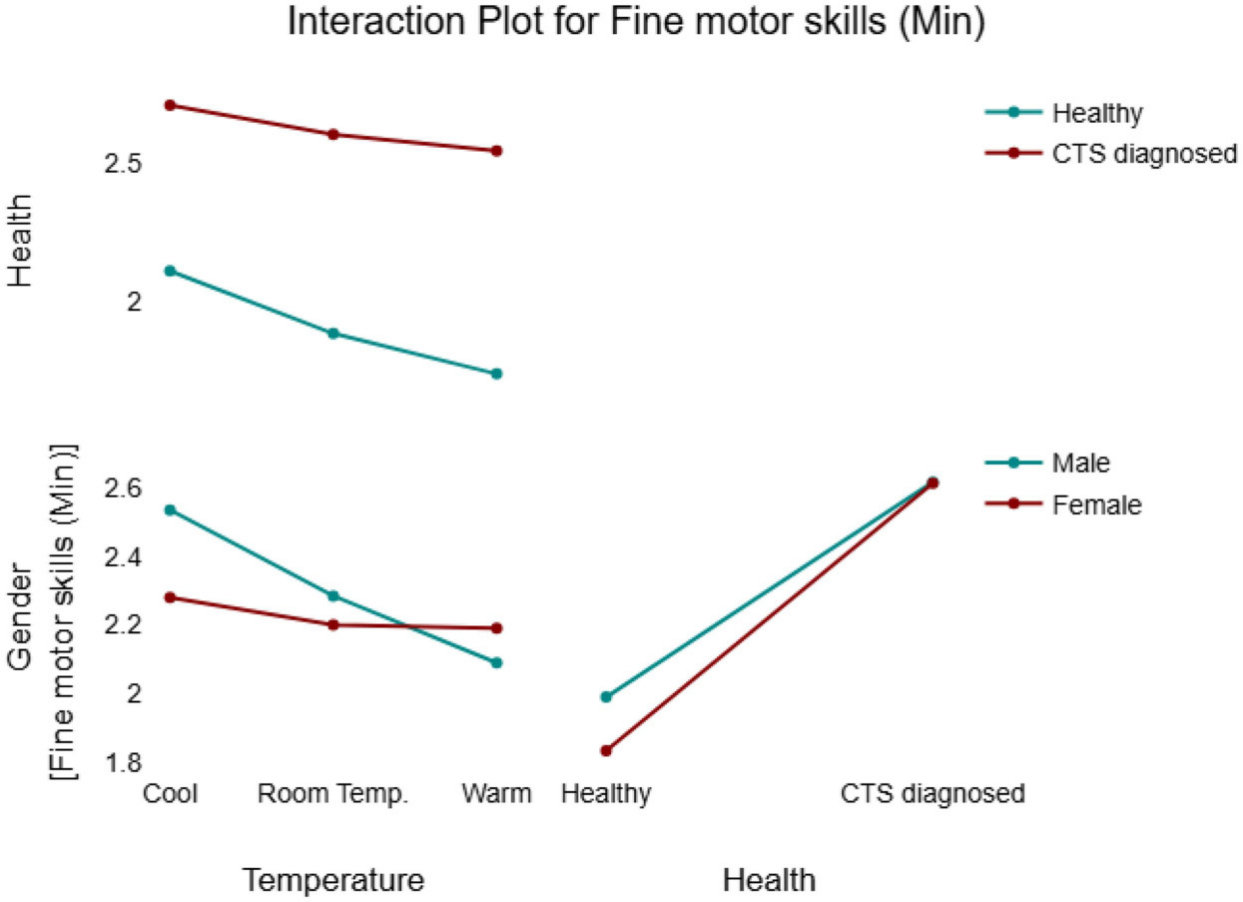
Interaction plot for fine motor skills model.

Two-way interactions presented an overview of how the relationship between a factor and a response would change if the level of another factor changes. In this model, no strong significant two-way interactions were observed except for Temperature(warm)*Gender. This significant two-way interaction (*p* = 0.042) indicates that males and females’ reaction to temperature change is different and can be evaluated in terms of task completion time. Hence, completion time of a male participant is expected to be 0.18 higher than a female participant at higher levels of temperature (see Figure 3).

### Gross motor skills

The Minnesota Dexterity Test was used to evaluate participants’ gross functional dexterity. The Shapiro–Wilk normality test with 95% confidence level has a *p*-value of 0.159 indicating that the model data does not significantly deviate from normality (20).

In terms of the analysis of variance, both temperature and gender were found to have a high significant effect on gross motor skills with *p*-values of 0.002 and 0.001 respectively. However, health had a very small and nonsignificant effect (f significance = 15.43) on gross motor skills. The fact that gross functional tests usually employ larger muscles and focus on arm movements rather than the hand and wrist justifies these results. Males with larger and stronger muscles scored significantly lower time to complete the test compared to females. Notably, the completion time of CTS diagnosed participants had no significant variation compared to healthy participants since their CTS symptoms (mainly in fingers and wrist) does not affect their gross motor skills.

A significant two-way interaction was found between temperature*health, suggesting that the healthy and the CTS groups do not respond similarly to changes in temperature. This may be attributed to the fact that individuals with CTS often have difficulty adapting to lower temperatures, as vasoconstriction (narrowing of blood vessels) can exacerbate CTS-related pain and further impair hand dexterity (17).

**Figure 4:**
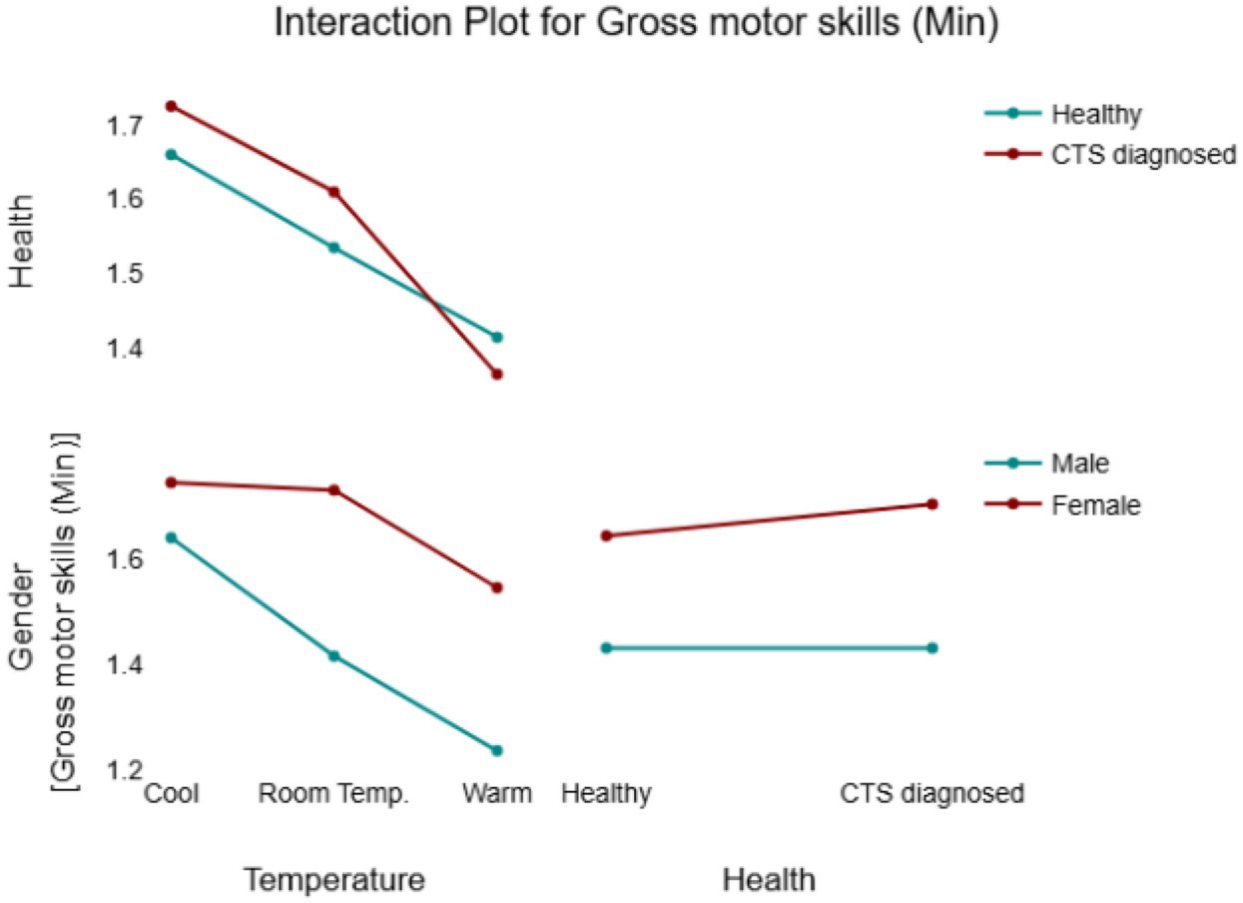
Interaction plot for the gross motor skills model.

A significant two-way temperature*gender interaction was found emphasizing that genders react differently to changes in temperature, and these differences become more noticeable at higher levels of temperature (see Table 5). To further explore this interaction, Figure 4 provides an overview of males vs. females behavior within each of the independent variables. When skin temperature increased from cool to room temperature, males demonstrated a 0.12 min shorter completion time compared to that of females. In other words, although both genders showed improved performance at higher temperatures, males outperformed females’ by approximately 0.12 min. When the temperature increased to the warm condition, this performance variation coefficient becomes 0.3 (see Table 6).

**Table 5:** ANOVA for the gross motor skills model.

Measure	DF	ADJ SS	ADJ MS	F	P
Model	9	0.41	0.05	258.42	.004
Temperature	2	0.19	0.09	530.33	.002
Health	1	0	0	15.43	.059
Gender	1	0.18	0.18	1,015.05	.001
Temperature*health	2	0.01	0	27.57	.035
Temperature*gender	2	0.03	0.01	82.05	.012
Health*gender	1	0	0	15.43	.059
Error	2	0	0		

**Table 6:** Estimated coefficients for the gross motor skills model.

Model	Coefficients	Standard error	t	*p*
Constant	1.69	0.01	255.88	<.001
Temperature_room temp.	−0.12	0.01	−12.83	.006
Temperature_warm	−0.3	0.01	−32.34	.001
Health	0.03	0.01	4.91	.039
Gender	0.05	0.01	7.94	.016
Temperature_room temp.*health	0	0.01	0.53	.646
Temperature_warm*health	−0.06	0.01	−6.15	.025
Temperature_room temp.*gender	0.11	0.01	11.22	.008
Temperature_warm*gender	0.1	0.01	10.96	.008
Health*Gender	0.02	0	3.93	.059

In addition, a significant Temperature*health interaction was identified, with a significant estimated coefficient of −0.06. Although a small variation in task performance was expected between healthy individuals and those diagnosed with CTS, the overall effect of health status on task completion time in the gross dexterity test was not statistically significant. Figure 4 provides a detailed overview of the interaction effects between the tested variables and the response.

**Figure 5:**
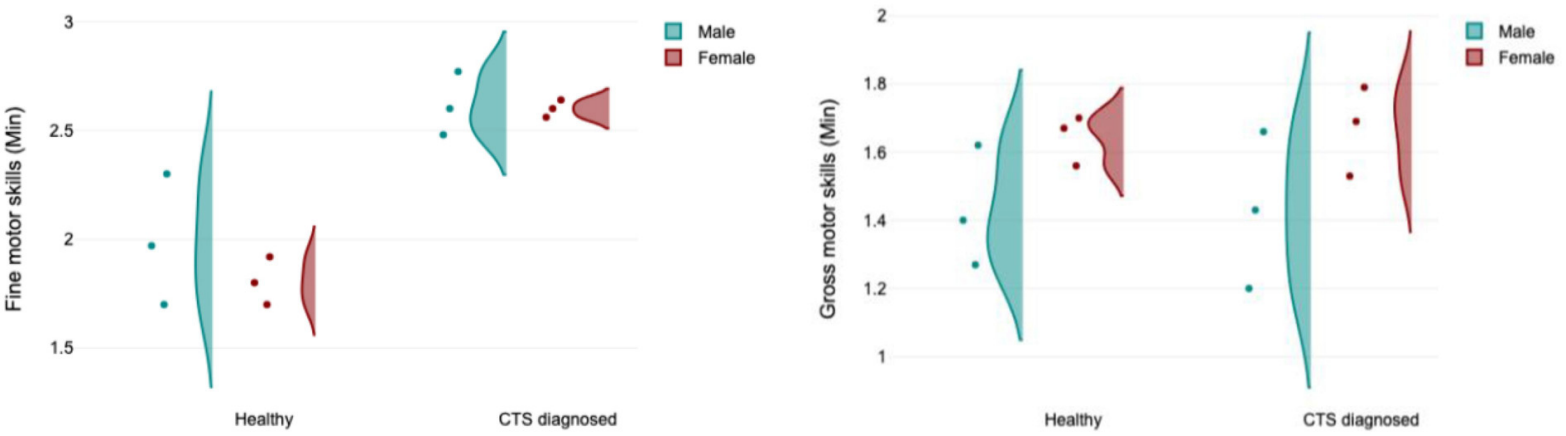
Raincloud plot compares results between fine motor skills test and gross motor skills test in term of gender and health.

The following figure (Figure 5) provides a comparison between fine motor skills and gross motor skills in terms of their dependence on health and gender. The main effect of gender is weak on fine motor skills, and it is stronger on gross motor skills. However, the main effect of health looks stronger on the fine motor skills plot compared to the gross motor skills plot.

### Hand grip strength (HGS)

The Shapiro–Wilk normality test with 95% confidence level was run to confirm normality assumption for this model. Shapiro–Wilk has *p*-value of 0.225 which indicates that the model data does not have significant deviation from normality (20).

**Figure 6:**
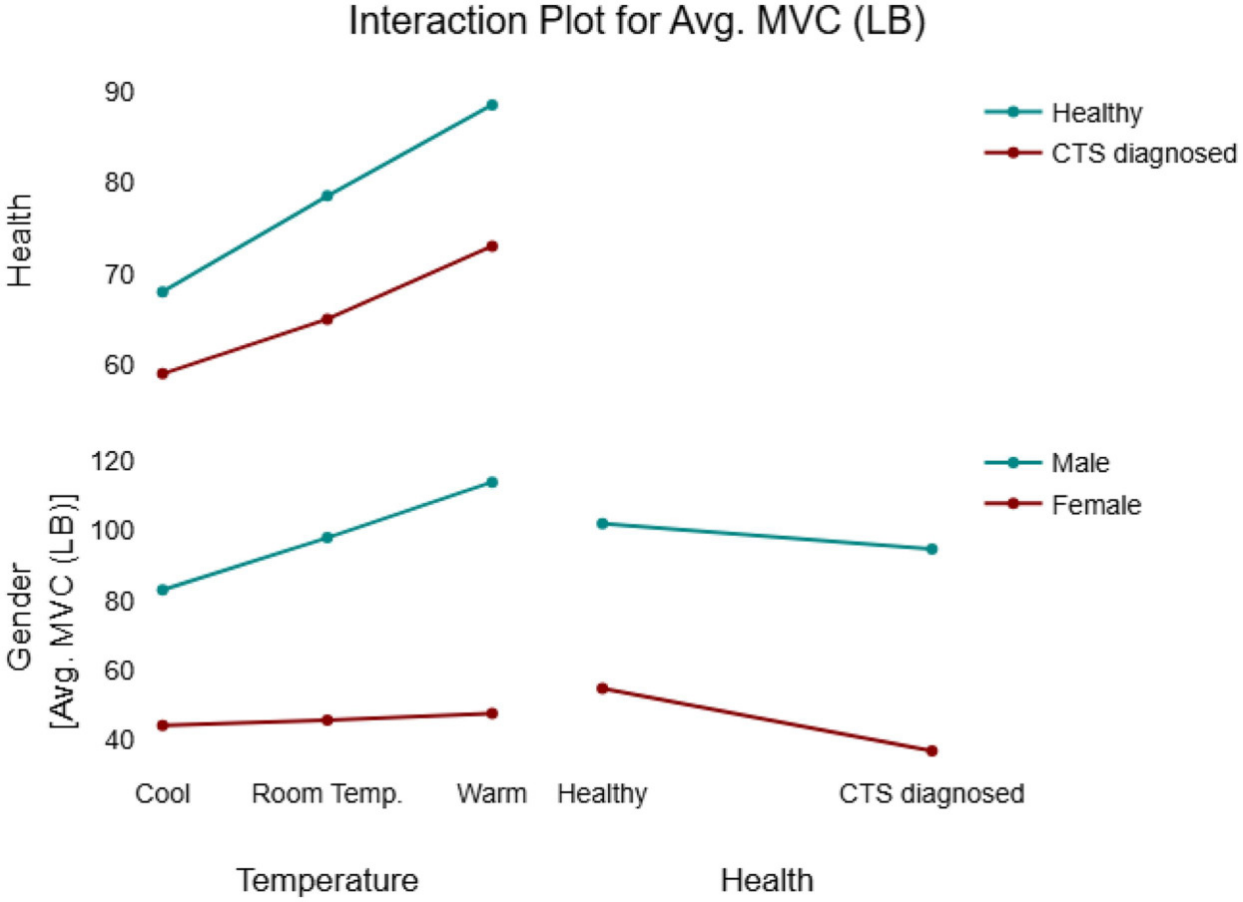
Interaction for the HGS model.

To evaluate the forearm muscles strength (HGS), a dynamometer was used to perform grip strength test in neutral position, supination and pronation, and the average value was calculated for each run. The ANOVA test showed a significant effect of temperature (*p* = 0.017), health (*p* = 0.01), and gender (*p* = 0.001) on the response HGS. According to Table 7, the average HGS is expected to increase with increasing skin temperature with a significant interaction between temperature and gender. Table 8 confirms the variation of gender performance at high temperature levels with a significant coefficient of 8.24 and 17.25 for room temperature level and warm level, respectively. Figure 6 emphasizes that female participant performance is less affected by changes in skin temperature since the value of slope is smaller compared to that of male participants.

**Table 7:** ANOVA for the HGS model.

Measure	df	Adj SS	Adj MS	F	*p*
Model	9	9,883.83	1,098.2	216.04	.005
Temperature	2	595.5	297.75	58.57	.017
Health	1	481.33	481.33	94.69	.01
Gender	1	8,321.33	8,321.33	1,636.98	.001
Temperature*health	2	22.17	11.08	2.18	.314
Temperature*gender	2	378.17	189.08	37.2	.026
Health*gender	1	85.33	85.33	16.79	.055
Error	2	10.17	5.08		

**Table 8:** Estimated coefficients for the HGS model.

Model	Coefficients	Standard error	t	*p*
Constant	63.5	1.13	56.33	<.001
Temperature_room temp.	8.25	1.59	5.17	.035
Temperature_warm	17.25	1.59	10.82	.008
Health	−4.5	1.13	−3.99	.057
Gender	−19.5	1.13	−17.3	.003
Temperature_room temp.*health	−2.25	1.59	−1.41	.294
Temperature_warm*health	−3.25	1.59	−2.04	.178
Temperature_room temp.*gender	−6.75	1.59	−4.23	.052
Temperature_warm*gender	−13.75	1.59	−8.62	.013
Health*gender	−2.67	0.65	−4.1	.055

### Effect sizes

Table 9 summarizes the Partial Eta-Squared (*η*_p_²) as a measure of effect size. The inclusion of effect size estimates underscores the practical significance of the observed findings, even for effects approaching the statistical significance threshold. The effect of health status (CTS) on fine motor skills (*η*_p_² = 0.984) and average MVC (*η*_p_² = 0.938) was very large, indicating that CTS is the predominant determinant of both motor precision and strength reduction. Similarly, gender exhibited a very large effect on average MVC (*η*_p_² = 0.993) and gross motor skills (*η*_p_² = 0.975), consistent with established physiological and anthropometric differences between males and females. Moreover, temperature demonstrated a very large effect on gross motor skills (*η*_p_² = 0.994), suggesting that thermal conditions exert a substantial influence on performance in tasks involving larger muscle groups. Collectively, these results highlight that the effects identified are not only statistically detectable but also clinically and functionally meaningful.

**Table 9:** Effect sizes of dependent variables.

Dependent Variable	Source	Partial eta squared (*η*_p_²)
Fine motor skills (min)	Health (CTS vs. healthy)	0.984
	Gender (male vs. female)	0.809
	Temp * gender	0.918
Gross motor skills (min)	Temperature (cool, room, warm)	0.994
	Health (CTS vs. healthy)	0.951
	Gender (male vs. female)	0.975
Avg. MVC (LB)	Health (CTS vs. healthy)	0.938
	Gender (male vs. female)	0.993
	Temp * gender	0.974

Table 10 reports the 95% Confidence Intervals for the means of the main effect levels, providing a measure of the precision of the estimates. The narrow CIs for the Fine Motor Skills and Gross Motor Skills means (especially for the Health and Gender factors) indicate a high degree of precision in these estimates. The wider CIs for Avg. MVC reflect the higher variability in strength measurements, which is expected.

**Table 10:** Confidence intervals for main effect means.

Factor	Level	Mean	Standard deviation	95% confidence interval
Fine motor skills (min)	CTS	2.615	0.093	[2.517, 2.713]
	Healthy	1.912	0.214	[1.687, 2.136]
	Female	2.223	0.430	[1.772, 2.675]
	Male	2.303	0.403	[1.880, 2.726]
Gross motor skills (min)	Cool	1.693	0.073	[1.577, 1.808]
	Room Temp.	1.573	0.189	[1.272, 1.873]
	Warm	1.390	0.182	[1.101, 1.679]
	Female	1.673	0.111	[1.557, 1.790]
	Male	1.430	0.184	[1.237, 1.623]
AVG. MVC (LB)	CTS	65.67	33.16	[30.86, 100.47]
	Healthy	78.33	27.98	[48.97, 107.69]
	Female	45.67	10.29	[34.87, 56.46]
	Male	98.33	14.45	[83.17, 113.49]

### sEMG signals

**Figure 7:**
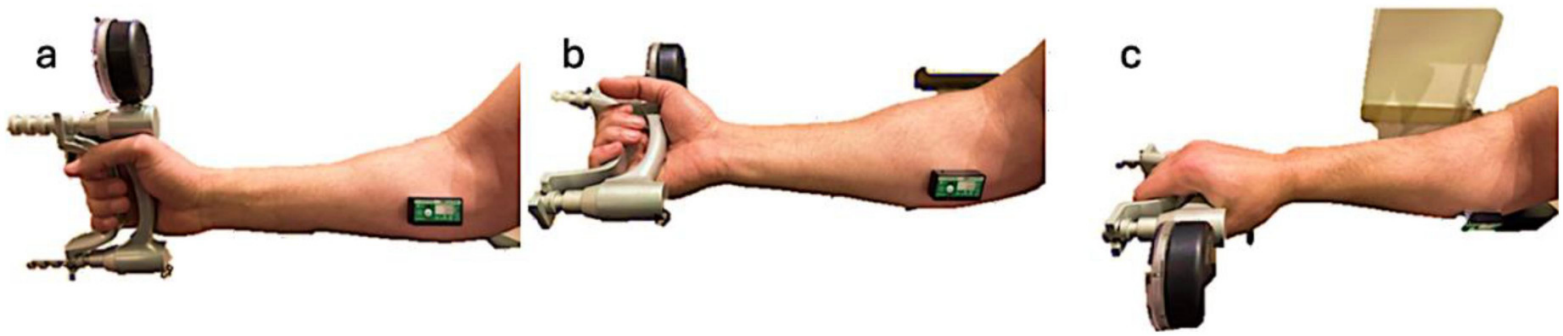
HSG test monitored with sEMG forearm sensor in three positions. **a**. Neutral position. **b**. Supination. **c**. Pronation.

Figure 7 shows forearm sensor (Biometrics K23866) which was installed at the forearm muscle belly to track electrical activity during HSG test in three positions: neutral, supination, and pronation. Surface electromyography (sEMG) signals provide information about muscle effort in terms of normalized Root Mean Square (nRMS) and mean frequency as a measure of the force exerted by the muscle and muscle fatigue (21).

In general, stronger muscles are able to perform stronger contractions, which means higher muscle activation. Stronger contractions require more motor units and higher firing rates leading to higher nRMS values. Variability is expected due to other variables including temperature and neuromuscular disorders such as CTS. Higher body temperature is typically associated with increased blood flow to the muscles, which promotes more efficient contractions. However, mean frequency can serve as an indicator of the muscle fatigue following repeated contractions. Shortly after muscle activation, higher mean frequencies are generally observed during stronger contractions. Over time, with prolonged muscle use, the mean frequency tends to decline due to the accumulation of metabolic byproducts (22–24). Table 11 provides the values of nRMS, and mean frequency calculated from the processed signals of the sEMG sensor. Spearman correlation analysis was run which showed a positive and significant correlation between *Avg.* Maximal voluntary contraction (*MVC)* and *nRMS* with *r*(10) = 0.7246, *p* = .008. Regarding mean frequency, no clear relationship with Avg. MVC was observed. This is due to the other factors affecting mean frequency at the recording time, including fiber composition, and muscle strength (training level) of the participant.

**Figure 8:**
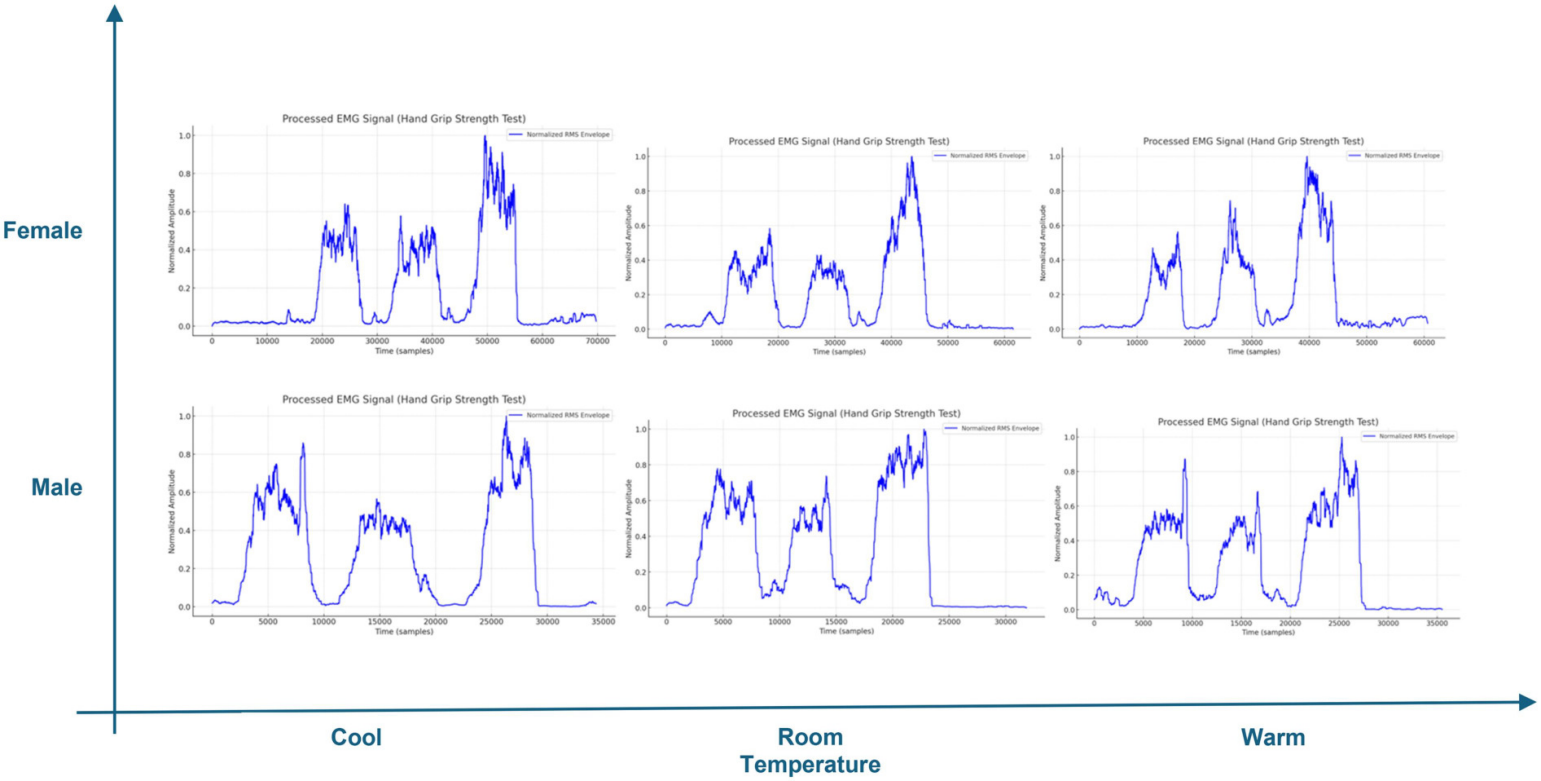
Processed sEMG signal of HGS test for healthy group.

**Figure 9:**
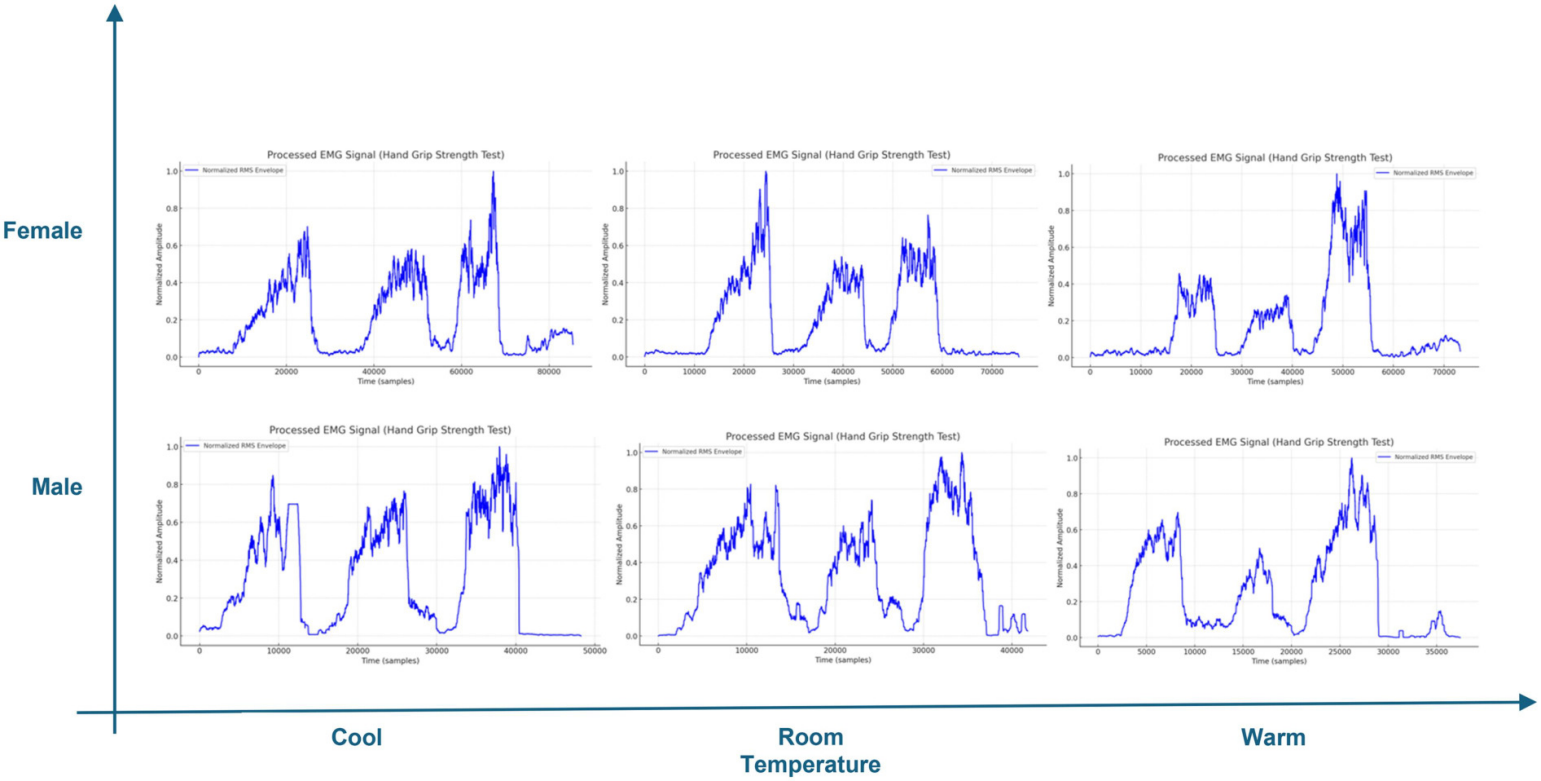
Processed sEMG signal of HGS test for CTS diagnosed group.

The next two figures (Figures 8, 9) include processed signals recorded by the forearm sEMG sensor during the HGS test. Raw signals were first filtered with Fast Fourier transform (FFT) to find cutoff frequencies due to sensor noise (in this case found to be 20–450 Hz), then a full wave rectification was done to absolute negative signals. The moving average of the original data was taken, and root mean square was calculated. Finally, signals were normalized and displayed as amplitude-time samples. Data processing was done using MATLAB 2019b. Separate figures are presented for healthy, and CTS diagnosed groups, both categorized by gender and temperature level. In general, healthy groups have narrower peaks compared to the CTS-diagnosed groups, indicating shorter time to reach MVC. However, the peak gets wider with lowering the temperature.

**Table 11:** nRMS and mean frequency for sEMG signals during HGS test.

No. of Run	Avg. MVC (Lbs)	nRMS	Mean frequency
1	86	0.383	3.23 Hz
2	102	0.429	3.14 Hz
3	118	0.425	3.11 Hz
4	80	0.354	4.04 Hz
5	94	0.368	3.95 Hz
6	110	0.413	3.29 Hz
7	50	0.295	3.61 Hz
8	55	0.303	4.54 Hz
9	59	0.282	3.70 Hz
10	38	0.302	4.53 Hz
11	36	0.296	3.96 Hz
12	36	0.368	4.69 Hz

## Discussion

This study explored the interplay between ambient temperature, gender, and musculoskeletal health on upper limb function, with a specific focus on dexterity and muscular strength. The results contribute to the growing body of evidence that ambient temperature is a key modulator of neuromuscular performance. In alignment with previous studies (2, 4), exposure to cold environments significantly reduced fine and gross motor dexterity as well as hand grip strength (HGS). This decline can be attributed to physiological responses such as vasoconstriction, decreased peripheral blood flow, and increased muscular stiffness, all of which impair neuromotor control and performance (6).

The findings also underscore the disproportionate impact of Carpal Tunnel Syndrome (CTS) on fine motor tasks. CTS-diagnosed participants exhibited significantly longer task completion times in the fine motor skills test compared to healthy controls. This aligns with the pathophysiology of CTS, which predominantly affects the fingers and wrist, resulting in impaired coordination and muscular fatigue (9). Conversely, gross motor tasks, which rely more heavily on larger proximal muscle groups, were not significantly affected by CTS status, suggesting a task-dependent vulnerability in individuals with neuromuscular disorders.

Gender emerged as another significant determinant of performance, particularly in gross motor skills and grip strength. As supported by existing literature (25), male participants consistently outperformed their female counterparts in strength and gross dexterity tasks, likely due to physiological differences in muscle mass and upper limb strength. Interestingly, female participants exhibited more stable performance across varying temperature conditions, which may suggest differences in thermoregulatory mechanisms or neuromuscular adaptability, a hypothesis that warrants further investigation.

The large gender differences in Avg. MVC and Gross Motor Skills (*η*_p_²) stem from more than body size. They reflect key physiological distinctions. Men's greater muscle mass and fiber size explain higher strength, while women's higher body fat and lower muscle mass affect thermoregulation, making them more sensitive to cold and more prone to performance drops in cool conditions. Though women often resist fatigue better in steady tasks, this advantage is less evident in strength-based movements. Overall, the results highlight that gender-related performance differences arise from intertwined effects of muscle strength, thermoregulation, and task type (26).

Electromyographic (sEMG) analysis supported these behavioral findings by revealing strong positive connection between average muscle force (MVC) and normalized root mean square (nRMS) values, particularly under warm conditions. This observation is consistent with prior studies highlighting the relationship between muscle activation, temperature, and contraction efficiency (21). Although the observed gender difference in dexterity scores (0.12) appears modest, it represents a consistent and functionally relevant variance in fine motor performance. Given that dexterity tasks are sensitive to neuromuscular control and occupational precision, such differences should not be disregarded when interpreting upper-limb performance or designing ergonomically balanced tools and workflows (27).

Together, these findings emphasize the complex interaction between individual characteristics and environmental conditions, with critical implications for optimizing work environments and minimizing health risks in temperature-variable settings.

### Limitations and future work

The sample size was relatively small (*N* = 12), which limits the statistical power and generalizability of the findings. However, full factorial design was used to examine all possible combinations of independent variables, supporting a comprehensive analysis of main effects and interactions with relatively few participants. The study's generalizability is limited by its young, healthy, CTS-diagnosed sample (aged 18–25), as upper limb strength, dexterity, and thermoregulation vary with age. Despite pilot testing to reduce learning effects, repeated dexterity trials across temperature conditions may still have introduced performance improvements unrelated to temperature. Additionally, high variability in Avg. MVC results suggests unmeasured other factors played a role such as pain tolerance, CTS severity, and physiological differences. Future research should include a broader age range, more extensive practice sessions, and objective measures of individual characteristics to improve accuracy and applicability.

## Conclusion

This study provides empirical evidence on how ambient temperature, musculoskeletal health, and gender collectively influence upper limb dexterity and strength. Key findings highlight a significant decline in motor performance under cold exposure and improved functional outcomes in warm conditions. Individuals diagnosed with CTS were particularly vulnerable to performance impairments, especially in fine motor tasks, likely due to compromised neuromuscular function. Furthermore, observed gender differences in muscular performance and temperature sensitivity underscore the need for personalized approaches in occupational task assignment.

These findings carry important implications for public health and workplace safety. Workers operating in cold environments, such as those in refrigerated storage, outdoor construction, or emergency services, may face increased risk of performance degradation, injury, and fatigue. The vulnerability is amplified for individuals with pre-existing neuromuscular conditions, underscoring the need for adaptive workplace strategies. Interventions may include environmental controls (e.g., localized heating), task rotation to reduce exposure time, gender- and health-informed job matching, and the provision of ergonomic equipment designed to maintain limb temperature and support muscular performance. By bridging experimental findings with practical applications, this study offers a more nuanced understanding of thermal ergonomics and supports the development of evidence-based guidelines aimed at improving worker safety, productivity, and well-being in thermally dynamic environments.

## Data Availability

The raw data supporting the conclusions of this article will be made available by the authors, without undue reservation.

## References

[1] Copaci D Pernalete N Ortiz AG Rojas DB. Proposed eye-hand correlation Assessment System: A Novel Approach for Evaluating Coordination. New York City: IEEE Access (2024).

[2] Ceballos D Mead K Ramsey J. Recommendations to improve employee thermal comfort when working in 40 F refrigerated cold rooms. J Occup Environ Hyg. (2015) 12(9):D216–21. 10.1080/15459624.2015.1047023 25961447 PMC4540649

[3] Imamura R Rissanen S Kinnunen M Rintamaki H. Manual performance in cold conditions while wearing NBC clothing. Ergonomics. (1998) 41(10):1421–32. 10.1080/001401398186180 9802250

[4] Orysiak J Młynarczyk M Irzmańska E. The impact of protective gloves on manual dexterity in cold environments—a pilot study. Int J Environ Res Public Health. (2022) 19(3):1637. 10.3390/ijerph19031637 35162660 PMC8835575

[5] Orysiak J Młynarczyk M Irzmańska E. The effect of exposure to cold on dexterity and temperature of the skin and hands. Int J Occup Saf Ergon. (2024) 30(1):64–71. 10.1080/10803548.2023.2293387 38191297

[6] Rall JA Woledge RC. Influence of temperature on mechanics and energetics of muscle contraction. Am J Physiol. (1990) 259(2):R197–203. 10.1152/ajpregu.1990.259.2.R197 2201213

[7] Rodrigues P Trajano GS Wharton L Orssatto LB Minett GM. A passive increase in muscle temperature enhances rapid force production and neuromuscular function in healthy adults. J Sci Med Sport. (2021) 24(8):818–23. 10.1016/j.jsams.2021.01.003 33487572

[8] Smith NP Barclay CJ Loiselle DS. The efficiency of muscle contraction. Prog Biophys Mol Biol. (2005) 88(1):1–58. 10.1016/j.pbiomolbio.2003.11.014 15561300

[9] Dabbagh A MacDermid JC Yong J Packham TL Macedo LG Ghodrati M. Diagnostic accuracy of sensory and motor tests for the diagnosis of carpal tunnel syndrome: a systematic review. BMC Musculoskelet Disord. (2021) 22:1–22. 10.1186/s12891-021-04202-y 33827512 PMC8028143

[10] Farioli A Curti S Bonfiglioli R Baldasseroni A Spatari G Mattioli S Observed differences between males and females in surgically treated carpal tunnel syndrome among non-manual workers: a sensitivity analysis of findings from a large population study. Ann Work Expo Health. (2018) 62(4):505–15. 10.1093/annweh/wxy015 29579135 PMC5905650

[11] Walter K. What is carpal tunnel syndrome? JAMA. (2022) 328(6):593–593. 10.1001/jama.2022.10522 35763267

[12] Flouris AD Cheung SS. Influence of thermal balance on cold-induced vasodilation. J Appl Physiol. (2009) 106(4):1264–71. 10.1152/japplphysiol.91426.2008 19213938

[13] Chang Y-W Chen C-J Wang Y-W Chiu V Lin S-K Horng Y-S. Influence of temperature on sonographic images of the median nerve for the diagnosis of carpal tunnel syndrome: a case control study. BMC Med Imaging. (2021) 21(1):163. 10.1186/s12880-021-00700-6 34742241 PMC8571853

[14] Frostadottir D Ekman L Zimmerman M Andersson S Arner M Brogren E Cold sensitivity, functional disability and predicting factors after a repaired digital nerve injury. Sci Rep. (2022) 12(1):4847. 10.1038/s41598-022-08926-2 35318398 PMC8941129

[15] Kadian-Dodov D. Cold hands or feet: is it raynaud’s or not? Medical Clinics. (2023) 107(5):829–44. 10.1016/j.mcna.2023.04.005 37541711

[16] McAlindon T Formica M Schmid CH Fletcher J. Changes in barometric pressure and ambient temperature influence osteoarthritis pain. Am J Med. (2007) 120(5):429–34. 10.1016/j.amjmed.2006.07.036 17466654

[17] Stjernbrandt A Vihlborg P Wahlström V Wahlström J Lewis C. Occupational cold exposure and symptoms of carpal tunnel syndrome–a population-based study. BMC Musculoskelet Disord. (2022) 23(1):596. 10.1186/s12891-022-05555-8 35725430 PMC9210706

[18] Bennett AF. Temperature and muscle. J Exp Biol. (1985) 115(1):333–44. 10.1242/jeb.115.1.333 3875678

[19] Farzadkia M Tajadini H Moghadam Ahmadi A Dehesh T Setayesh M. Efficacy of local heat therapy in alleviating symptoms of mild to moderate idiopathic carpal tunnel syndrome. Int Arch Health Sci. (2024) 11(2):79–84. 10.48307/iahsj.2024.411222.1042

[20] Shapiro SS Wilk MB. An analysis of variance test for normality (complete samples). Biometrika. (1965) 52(3–4):591–611. 10.1093/biomet/52.3-4.591

[21] Garcia MC Vieira T. Surface electromyography: why, when and how to use it. Revista Andaluza de Medicina del Deporte. (2011) 4(1):17–28.

[22] Ahn AN. How muscles function–the work loop technique. J Exp Biol. (2012) 215(7):1051–2. 10.1242/jeb.062752 22399648

[23] Allen DG. Fatigue in working muscles. J Appl Physiol. (2009) 106(2):358–9. 10.1152/japplphysiol.91599.2008 19095748

[24] Monod H. How muscles are used in the body. The structure and function of muscle. Structure. (2013) 1:23–74.

[25] Samodra YTJ Yosika GF Gustian U Mashud M Arifin S Suryadi D Are boys and girls in rural areas equal in terms of gross motor skills? Retos. (2024) 54:94–9. 10.47197/retos.v54.103005

[26] Hunter SK Senefeld JW. Sex differences in human performance. J Physiol (Lond). (2024) 602(17):4129–56. 10.1113/JP284198 39106346

[27] Desrosiers J Hebert R Bravo G Dutil E. Upper-extremity motor co-ordination of healthy elderly people. Age Ageing. (1995) 24(2):108–12. 10.1093/ageing/24.2.108 7793331

